# Quality of Vision after Deep Anterior Lamellar Keratoplasty (Fluid Dissection) Compared to Penetrating Keratoplasty for the Treatment of Keratoconus

**DOI:** 10.1155/2017/4507989

**Published:** 2017-07-20

**Authors:** Islam Mahmoud Hamdi, Momen Mahmoud Hamdi

**Affiliations:** ^1^Ophthalmology Department, Faculty of Medicine, Ain Shams University, Cairo, Egypt; ^2^The Eye Consultants Center, Jeddah, Saudi Arabia

## Abstract

**Purpose:**

To compare the visual quality of patients with keratoconus who underwent penetrating keratoplasty (PKP) or deep anterior lamellar keratoplasty (DALK) with fluid dissection.

**Design:**

Cross-sectional, observational study.

**Methods:**

Twelve eyes that underwent PKP (PKP group) were compared to 24 eyes that underwent DALK (DALK group) after complete removal of sutures and stability of refraction. Visual, refractive, corneal topographic, corneal aberrometry, and ocular aberrometry parameters were compared for both groups. The *χ*^2^ and Mann–Whitney *U* tests were used for comparisons as appropriate. *P* < 0.05 was considered statistically significant.

**Results:**

Uncorrected and best spectacle-corrected visual acuity (UCVA and BSCVA, resp.), mean refractive spherical equivalent and mean refractive cylinder (MRSE and MRC, resp.), root mean square of the 3 mm and 5 mm OPD Scan (NIDEK Co. Ltd., Gamagori, Japan), steep and flat meridians (SimK1 and SimK2, resp.), and the difference (corneal cylinder) were not statistically significantly different between groups (*P* > 0.05, all comparisons). All aberrations, point spread functions (PSF), and the modulation transfer function (MTF) were not statistically different between groups (*P* > 0.05).

**Conclusion:**

For our small study, the postoperative PKP and DALK with fluid dissection patient groups had vision/optical quality parameters that were not statistically different. This may indicate that DALK with fluid dissection can replace PKP for keratoconus without compromising vision quality.

## 1. Introduction

Over the last decade, deep anterior lamellar keratoplasty (DALK) has increasingly been advocated as a reliable alternative to penetrating keratoplasty (PKP) for the treatment of purely stromal diseases such as keratoconus [1, 2]. The advantages of DALK over PKP have been well documented including the mitigation of endothelial rejection, the reduced duration of postoperative immunosuppressive agents, and earlier suture removal [2]. However, PKP is a standardized technique yet the approach to DALK varies depending on surgeon preference. For example, DALK with manual dissection, fluid dissection and big bubble technique are technically different. Considerable residual stroma or baring of Descemet's membrane may occur depending on the DALK technique. The differing techniques may lead to a lack of predictability in visual outcomes.

Higher-order aberrations (HOA) can affect visual acuity and visual quality [3, 4]. HOA measures are routinely used for assessing postoperative objective visual quality of a number of ophthalmic procedures [5, 6]. Despite the advantages of DALK over PKP, the potential for reduced visual performance represents a significant drawback. Comparisons of HOA after PKP and DALK are rare [3, 7–9]. In previous publications [10, 11], Amayem et al. published the technique of DALK with fluid dissection, where the authors of this study participated partially [11] and reported the refractive outcomes of DALK versus PKP [10, 11]. In this study, we compare the postoperative objective visual quality after PKP or DALK with fluid dissection.

## 2. Patients and Methods

This is a cross-sectional, comparative study of consecutive patients with keratoconus who underwent penetrating keratoplasty (PKP group) or deep anterior lamellar keratoplasty with fluid dissection (DALK group). The study followed the tenets of the Declaration of Helsinki. A prior institutional review board approval was not required for the study. All surgeries were performed by one surgeon (IH). The clinical criteria for PKP for keratoconus included patients who had a steepest *K* value > 60.0 D and were hard contact lenses intolerant. DALK was performed on cases with an intact Descemet's membrane, whereas PKP was reserved for posthydrops cases. The surgical techniques have been previously described [10, 11]. Patients who experienced intra- or postoperative complications were excluded from comparison.

**Figure 1:**
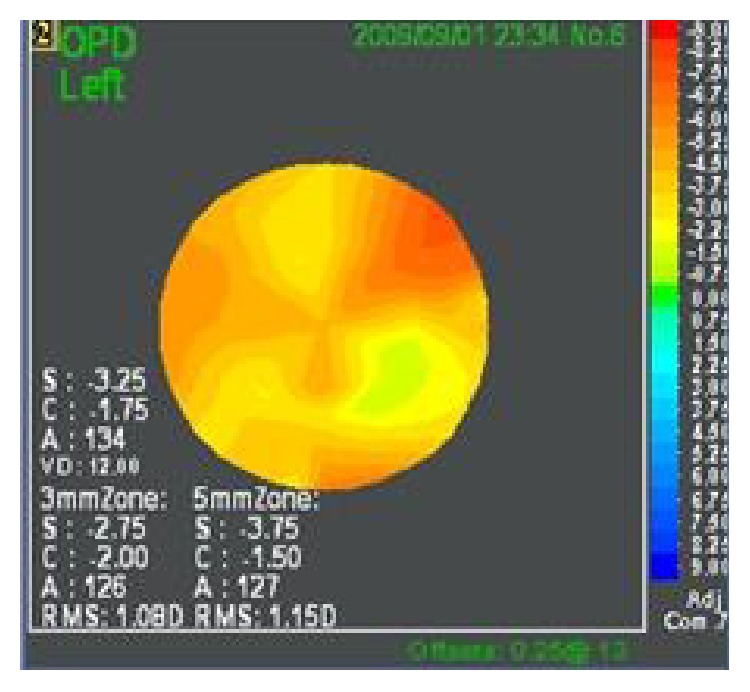
OPD Scan map demonstrating RMS of refraction at 3 and 5 mm.

For all cases, a reliable OPD Scan (NIDEK Co. Ltd., Gamagori, Japan) examination was performed, 2 months after complete removal of sutures (stability of refraction) and prior to any surgical correction of the residual refractive error. A reliable OPD Scan examination consisted of a well-centered corneal topographic map, corneal aberrometry, and ocular aberrometry that was free of artifacts due to small pupil size, lacrimal lake, and dry eye. Uncorrected and best spectacle corrected visual acuity (UCVA and BSCVA, resp.) were recorded in LogMAR notation for statistical comparison. Mean refractive spherical equivalent and mean refractive cylinder (MRSE and MRC, resp.) were compared. Homogeneity of refraction across the pupil was assessed as the root mean square (RMS value in diopters) of the refractions at 3 mm and 5 mm on the OPD Scan and compared between groups. The higher the RMS value at 3 mm or 5 mm, the greater the difference in refractive power (less homogenous or uniform refraction) at the corresponding diameter. Refraction was recorded from the OPD Scan (Figure 1). The steepest (Sim K1) and flattest (Sim K2) corneal meridians and the difference between these meridians (Kcyl) were compared between groups.

**Figure 2:**
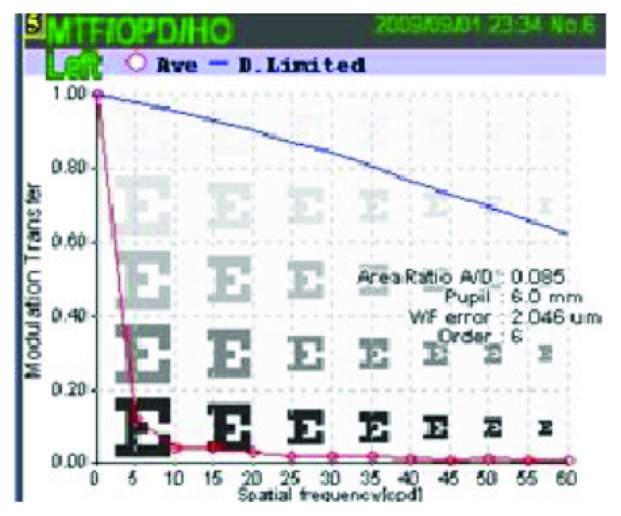
MTF graph demonstrating A/D.

Whole eye (ocular) and corneal wavefront aberrations were compared for a 6 mm entrance pupil to the 6th Zernike order. The RMS values (*μ*m) were evaluated for total high-order aberrations (HOA), coma, trefoil, tetrafoil, spherical like, and high-order astigmatism (HOAST). The Strehl ratio of point spread function (PSF) was used as an objective measure of glare. The modulation transfer function (MTF) was used as an objective measure of contrast sensitivity. A metric for the MTF is provided in the OPD Scan as an A/D value. A/D is the ratio of the area under the curve of the actual eye (A) and the area under the curve of a diffraction limited curve (D) (best optical system possible) (Figure 2), the higher the A/D value, the better the objective visual quality of the eye. PSF and MTF are reported for HOA only.

### 2.1. Statistical Analysis

Statistics were performed using SPSS software, version 12 (SPSS Inc., Chicago, IL, USA). Descriptive analysis was performed by calculating mean ± standard deviation and range for quantitative data. For qualitative data, frequencies were represented by a number and percentage. For parametric values, a between-group comparison was performed with the Student *t*-test for quantitative data and with *χ*^2^ test for qualitative data. For nonparametric values, the Mann–Whitney *U* test was used for between-group comparison. *P* < 0.05 was considered statistically significant.

## 3. Results

The study cohort comprised 36 consecutive eyes of 36 patients. Twelve eyes had undergone PKP (PKP group), and 24 eyes had undergone DALK (DALK group). Both groups were well matched in terms of age, gender, and mesopic pupil diameters (*P* > 0.05) (Table 1).

There were no statistically significant differences in UCVA, BSCVA, refraction (MRSE and MRC), homogeneity of refraction (RMS 3 mm and 5 mm), and topography (SimK1, SimK2, and Kcyl) between groups (*P* > 0.05 all comparisons) (Table 2).

RMS values of all corneal and ocular HOA were not statistically different between groups. Ocular and corneal Strehl ratio and MTF were not statistically significantly different between groups (*P* > 0.05, all comparisons) (Tables 3 and 4).

## 4. Discussion

In this comparison of objective visual quality after PKP or DALK (hydrodissection), we found that post-DALK eyes were performing as well as post-PKP eyes. The refractive and visual outcomes were similar between groups (*P* > 0.05, all comparisons).

The RMS of refraction across the pupil was similar between groups. In both groups, the RMS of refraction increased with increased pupil diameter indicating less uniformity with a mesopic pupil. This outcome was likely a byproduct of corneal surface irregularity. Our study showed (although statistically nonsignificant) more myopic refraction values in the DALK group supported by higher *K* values on corneal topography. This finding is supported by the results of a previous study by the same group on another sample of cases [11]. The statistically similar refractive and visual outcomes in our study concur with outcomes in the literature. In a similar comparative study, Javadi and colleagues [8] reported that the refractive and visual outcomes were not significantly different between groups. Similarly, Sögütlü and colleagues [3] compared DALK to PKP for keratoconus and found no statistical difference in postoperative refraction or vision between groups. Of note, refraction and vision were similar despite the differing DALK techniques used in the current study (fluid dissection) and those of Javadi and colleagues [8] and Sögütlü and colleagues [3] (big bubble). A thorough review of the literature by the American Academy of Ophthalmology concluded that refractive outcomes and best spectacle-corrected vision are similar between DALK and PKP [2].

The changes in HOA differ from those of Javadi and colleagues [8] who found lower ocular spherical aberration in the PKP group and lower fifth order aberrations in the DALK group. The different DALK techniques between surgeons may explain the difference between studies. The changes in HOA in the current study could be clinically significant as a change in RMS of wavefront error of 0.10 *μ*m is considered clinically meaningful [12]. However, our personal experience (no study data) indicates that 0.30 *μ*m RMS change or higher may be a better indicator of clinically significant change. Confirming the wavefront results, the objective visual quality did not differ between groups as determined by the Strehl ratio and modulation transfer function. MTF was used as an objective assessment for contrast sensitivity. Previous comparisons of contrast sensitivity are inconsistent. A recent study reported similar outcomes for post-PKP eyes compared to post-DALK eyes that underwent a Descemet's baring technique for DALK [13]. However, another study reported better mesopic contrast sensitivity at 3 cycles per degree in the DALK group compared to the PKP group despite similar levels of postoperative HOA [3].

The residual stromal bed in DALK technique seems to have an effect on the optical and perhaps postoperative visual quality [8]. Descemet's baring is expected to leave a smoother optical interface resulting in less optical aberrations [8]. Some have postulated that considerable light scatter at the interface may be detrimental to the optical quality of the eye [14]. If so, this makes a case for smoother interface and baring of Descemet's membrane during DALK. In this technique, there is no intension of baring Descemet's membrane. In contrary, the thinnest layer left is expected to be optically negligible and at the same time leaves a protection to Descemet's membrane, lowering the rate of perforation (a direct comparative study is needed to confirm this). Fluid is injected, hydrating stromal lamellae and adding a grayish tinge. Stroma is dissected completely as 2–4 layers. As a technical remark, the desired level of dissection is reached when the remaining stroma is transparent with a more or less smooth surface. It is expected that this level is just above the recently described Dua's layer [15]. Still, the residual stromal thickness is unpredictable, immeasurable, and variable [11]. However, once mastered, consistency of the results is remarkably appreciated. This is confirmed by the results of this study. Although, never estimated (intra- or postoperatively), the residual layer did not affect the final result.

The small study size represents a limitation of this study. Another limitation was the difference in photopic pupil diameter. However, the impact of mesopic pupil diameter is generally higher and we expect this difference would have little effect on the outcomes.

In conclusion, the thinner residual stroma after the DALK using fluid dissection did not sacrifice the quality of vision compared to PKP. The refractive and visual outcomes were similar between groups. Comparison between techniques of DALK and optical effect in other purely stromal diseases warrants investigation.

## Figures and Tables

**Table 1:** Comparison of consecutive eyes that underwent deep anterior lamellar keratoplasty (fluid dissection) or penetrating keratoplasty for keratoconus.

	PKP	DALK	Test	*P*
Age (years)	26.95 ± 9.48	23.79 ± 5.96	*t*	>0.05
(Mean ± SD range)	(14–43)	(14–36)		

Gender	9/3	14/10	*χ* ^2^	>0.05
(M/F)	(75%–25%)	(58.35%–41.65%)		

Photopic pupil (mm)	4.46 ± 0.34	4.07 ± 0.65	*t*	<0.05
(Mean ± SD range)	(3.68–4.98)	(3.22–5.63)		

Mesopic pupil (mm)	6.39 ± 0.92	6.46 ± 0.91	*t*	>0.05
(Mean ± SD range)	(5.01–7.78)	(5.27–8.58)		

M = male; F = female; DALK = deep anterior lamellar keratoplasty (fluid dissection); PKP = penetrating keratoplasty; *t* = *T*-test; *χ*^2^ = Chi squared test; SD = standard deviation. *P* < 0.05 is statistically significant.

**Table 2:** Postoperative comparison of vision and refractive parameters of consecutive eyes that underwent deep anterior lamellar keratoplasty (fluid dissection) or penetrating keratoplasty for keratoconus.

	PKP Mean ± SD (min–max)	DALK Mean ± SD (min–max)	Test	*P*
UCVA	0.88 ± 0.55	0.86 ± 0.47	*t*	>0.05
(LogMAR)	(0.2–1.5)	(0–1.5)		

BSCVA	0.18 ± .18	0.2 ± 0.21	*U*	>0.05
(LogMAR)	(0–0.7)	(0-1)		

MRSE	−2.65 ± 3.2	−4.31 ± 3.55	*U*	>0.05
(D)	(−8–5)	(−11.0–3.25)		

MRC	−3.96 ± 2.93	−3.99 ± 2.93	*U*	>0.05
(D)	(0–10.5)	(0–7.5)		

RMS 3 mm	1.46 ± 0.72	0.91 ± 0.41	*t*	>0.05
(D)	(0.52–3.15)	(0.21–1.55)		

RMS 5 mm	2.46 ± 1.53	1.7 ± 0.84	*U*	>0.05
(D)	(0.89–6.39)	(0.42–3.15)		

SimK1	46.16 ± 2.43	48.32 ± 2.68	*t*	>0.05
(D)	(43.38–51.53)	(43.49–53.07)		

SimK2	41.74 ± 2.29	43.86 ± 2.66	*t*	>0.05
(D)	(36.93–44.41)	3(9.2–48.98)		

Kcyl	4.42 ± 3.07	4.31 ± 2.76	*U*	>0.05
(D)	(0.89–11.71)	(1.31–9.94)		

UCVA = uncorrected visual acuity; BSCVA = best spectacle-corrected visual acuity; MRSE = mean refractive spherical equivalent; MRC = mean refractive cylinder; SimK1 = simulated *K* value at steep meridian; SimK2 = simulated *K* value at flat meridian; Kcyl = difference between steep and flat *K* values; RMS = root mean square; DALK = deep anterior lamellar keratoplasty (fluid dissection); PKP = penetrating keratoplasty; wavefront values are presented in *t* = *T*-test and *U* = Mann–Whitney *U* test. *P* < 0.05 is statistically significant.

**Table 3:** Postoperative comparison of ocular (whole eye) aberrometry and optical quality of consecutive eyes that underwent deep anterior lamellar keratoplasty (fluid dissection) or penetrating keratoplasty for keratoconus.

Aberrations	PKP Mean ± SD (min–max)	DALK Mean ± SD (min–max)	Test	*P*
HOA	1.73 ± 1.13	1.59 ± 0.61	*t*	>0.05
(*μ*m)	(1.018–4.725)	(0.173–2.763)		

Coma	1.35 ± 0.99	0.92 ± 0.52	*U*	>0.05
(*μ*m)	(0.122–3.805)	(0.126–1.752)		

Trefoil	1.27 ± 1.29	0.97 ± 0.46	*U*	>0.05
(*μ*m)	(0.236–3.908)	(0.057–1.913)		

Tetrafoil	0.45 ± 0.25	0.41 ± 0.19	*t*	>0.05
(*μ*m)	(0.159–0.973)	(0.111–0.79)		

Spherical like	0.55 ± 0.28	0.52 ± 0.35	*U*	>0.05
(*μ*m)	(0.127–0.867)	(0.061–1.325)		

HOAst	0.33 ± 0.11	0.27 ± 0.1	*t*	>0.05
(*μ*m)	(0.127–0.498)	(0.069–0.486)		

PSF	4.08*e*−03 ± 2.84*e*−03	7.92*e*−03 ± 8.34*e*−03	*U*	>0.05
(Strehl ratio)	(0.001–0.01)	(0.002–0.042)		

MTF	8.39*e*−02 ± 1.81*e*−02	0.1 ± 3.06*e*−02	*t*	>0.05
(A/D)	(0.065–0.121)	(0.075–0.193)		

HOA = higher-order aberrations; HOAst = high-order astigmatism; PSF = point spread function; MTF = modulation transfer function; DALK = deep anterior lamellar keratoplasty (fluid dissection); PKP = penetrating keratoplasty; *t* = *T*-test; *U* = Mann–Whitney *U* test. *P* < 0.05 is statistically significant.

**Table 4:** Postoperative comparison of corneal aberrometry and optical quality of consecutive eyes that underwent deep anterior lamellar keratoplasty (fluid dissection) or penetrating keratoplasty for keratoconus.

Aberrations	PKP Mean ± SD (min–max)	DALK Mean ± SD (min–max)	Test	*P*
HOA	3.24 ± 1.27	2.27 ± 0.96	*t*	>0.05
(*μ*m)	(1.407–5.8)	(0.191–4.01)		

Coma	1.98 ± 1.51	1.67 ± 0.82	*U*	>0.05
(*μ*m)	(0.249–5.635)	(0.206–3.057)		

Trefoil	1.73 ± 1.1	1.48 ± 0.39	*t*	>0.05
(*μ*m)	(0.292–3.47)	(0.325–1.708)		

Tetrafoil	0.51 ± 0.22	0.58 ± 0.34	*t*	>0.05
(*μ*m)	(0.23–1.03)	(0.022–1.327)		

Spherical-like	1.04 ± 0.39	1.09 ± 0.58	*t*	>0.05
(*μ*m)	(0.143–1.592)	(0.173–2.502)		

HOAst	0.46 ± 0.21	0.36 ± 0.33	*U*	>0.05
(*μ*m)	(0.07–0.801)	(0.078–1.628)		

PSF	3.17*e*−03 ± 1.99*e*−03	5.17*e*−03 ± 5.86*e*−03	*U*	>0.05
(Strehl ratio)	(0.001–0.008)	(0.001–0.027)		

MTF	7.6*e*−02 ± 1.01*e*−02	8.52*e*−02 ± 2.09*e*−02	*t*	>0.05
(A/D)	(0.063–0.1)	(0.067–0.147)		

HOA = higher-order aberrations; HOAst = high-order astigmatism; PSF = point spread function; MTF = modulation transfer function; DALK = deep anterior lamellar keratoplasty (fluid dissection); PKP = penetrating keratoplasty; *t* = *T*-test; *U* = Mann–Whitney *U* test. *P* < 0.05 is statistically significant.
